# Circulating tumor cells in colorectal cancer in the era of precision medicine

**DOI:** 10.1007/s00109-021-02162-3

**Published:** 2021-11-20

**Authors:** Mingchao Hu, Zhili Wang, Zeen Wu, Pi Ding, Renjun Pei, Qiang Wang, Chungen Xing

**Affiliations:** 1 Department of Gastrointestinal Surgery, The Second Affiliated Hospital of Soochow University, Suzhou, 215000 China; 2 CAS Key Laboratory of Nano-Bio Interface, Suzhou Institute of Nano-Tech and Nano-Bionics, Chinese Academy of Sciences, Suzhou, 215123 China; 3 Department of General Surgery, The Affiliated Jiangsu Shengze Hospital of Nanjing Medical University, Suzhou, 215228 China

**Keywords:** Biomarkers, Circulating tumor cells, Colorectal cancer, Precision medicine

## Abstract

Colorectal cancer (CRC) is one of the main causes of cancer-related morbidity and mortality across the globe. Although serum biomarkers such as carcinoembryonic antigen (CEA) and carbohydrate antigen 19–9 (CA-199) have been prevalently used as biomarkers in various cancers, they are neither very sensitive nor highly specific. Repeated tissue biopsies at different times of the disease can be uncomfortable for cancer patients. Additionally, the existence of tumor heterogeneity and the results of local biopsy provide limited information about the overall tumor biology. Against this backdrop, it is necessary to look for reliable and noninvasive biomarkers of CRC. Circulating tumor cells (CTCs), which depart from a primary tumor, enter the bloodstream, and imitate metastasis, have a great potential for precision medicine in patients with CRC. Various efficient CTC isolation platforms have been developed to capture and identify CTCs. The count of CTCs, as well as their biological characteristics and genomic heterogeneity, can be used for the early diagnosis, prognosis, and prediction of treatment response in CRC. This study reviewed the existing CTC isolation techniques and their applications in the clinical diagnosis and treatment of CRC. The study also presented their limitations and provided future research directions.

## Introduction

Despite recent advancements, colorectal cancer (CRC) is still one of the leading causes of cancer-related deaths worldwide. With 1.65 million new cases and about 835,000 deaths annually, CRC is ranked as the third most prevalent cancer globally and second in terms of mortality [1, 2]. The 5-year survival rate of CRC is as high as 90% when diagnosed at stage I or II, while it decreases to 13% when diagnosed at stage III or IV [1]. Although some patients can be cured by radical surgery, patients with heavy tumor burden, neurovascular invasion, and even distant metastasis exhibit an extremely poor prognosis. Carcinoembryonic antigen (CEA) is an established biomarker with reported efficacy for the treatment and monitoring of human cancers. It is the only biomarker recommended for monitoring and evaluating the prognosis of CRC in the current treatment guidelines [3]. Several blood tumor markers, including CA72-4 and CA19-9, are known to have limited sensitivity and specificity [4]. The identification of novel noninvasive biomarkers in plasma for diagnostic and prognostic purposes is thus of critical importance.

Tumor biopsies are now regarded as the “gold standard” in the diagnosis, prognosis, and prediction of therapy response in treating patients with cancers [5]. However, cancer cells are highly heterogeneous at the single-cell level, and biopsy specimens containing a small amount of tumor tissue may not represent entire cancer as a whole [6]. Besides, in the course of disease progression, cancers may have escape mutations and epigenetic alterations as a result of dynamic molecular changes [7, 8], which may also contribute to the cancer progression and resistance to therapy. Nevertheless, it is impractical to perform repeat tumor tissue biopsies during the patient’s illness. Just as colonoscopy, while tumor biopsy can be used to examine the entire colon and diagnose CRC, it is also an invasive technique that can cause complications such as bleeding, perforation, and cardiovascular accidents [9–11]. Not only are new biomarkers needed to evaluate the prognosis, treatment, and recurrence of CRC, but the search for these biomarkers with new minimally invasive techniques is also essential.

Compared with traditional tissue biopsy, liquid biopsy is a revolutionary technique that presents a static snapshot of the tumor and offers unique and enormous advantages. It provides information on the cancer burden in “real time” and unveils the evolution and heterogeneity of the disease over time [12]. The main targets of liquid biopsy include circulating tumor cells (CTCs), circulating tumor DNA (ctDNA), and extracellular vesicles. Tumor cells that enter the bloodstream from primary or metastatic cancers are called CTCs, the concept of which was first proposed by Ashworth at the end of the nineteenth century [13–15]. The abundance of CTCs in peripheral blood is incredibly low, with approximately 1 CTC per 10^6^–10^7^ white blood cells. Furthermore, the frequency of CTCs is even lower when solid tumors are confined to local growth [16], making the detection and isolation of CTCs extremely difficult and challenging. However, these rare cells can provide a wealth of tumor-related clinical information. Prompting the development of more sensitive platforms for isolating, enriching, and investigating CTCs can provide a noninvasive alternative for diagnosing, monitoring treatment, and evaluating the prognosis of malignant tumors. In-depth analysis of CTCs provides useful information for investigating the molecular characteristics of cancers, early detection of primary and metastatic lesions, and personalized treatment, mainly in terms of prognostic evaluation, stratified targeting of patients and real-time monitoring of treatment effects, identification of treatment targets, and drug resistance mechanisms [17]. This review presented the development of CTC isolation methods and their clinical applications in CRC and discussed their feasibility as potential biomarkers for the diagnosis and prognosis of CRC.

## Overview of the biological and clinical significance of CTCs

During tumor growth, individual or clusters of tumor cells detach from the primary site and pass through the blood vessels, eventually entering the circulation and forming “circulating tumor cells.” Moreover, tumor cells may move through channels formed by the proteolysis of other tumor cells or pre-existing channels formed by tissue structures [15]. In fact, most CTCs released in the bloodstream die in an early stage due to the combined effects of mechanical and environmental factors such as shear stress, oxidative stress, and immune system response [18]. As a result, the half-life of CTCs in the cycle is noticeably short, usually between 1 and 2 h [19], and only a few drug-resistant cells can extravasate and spread. CTCs must undergo a series of adaptations to survive in the changing environment. The most important of these adaptations is the process of epithelial-to-mesenchymal transition (EMT), which allows tumor cells to reduce epithelial characteristics, limit polarity, and promote the transformation of mesenchymal phenotypes, characterized by higher migratory and invasive potential [20–23]. The activation of EMT in cancer cells has important functional consequences, given that mesenchymal tumor cells are more aggressive and resistant to treatment [24]. The proportion of mesenchymal CTCs increases with disease progression, eventually resulting in distant metastasis [25]. Over the recent years, it has been suggested that tumor cells must undergo a reverse mesenchymal-to-epithelial transition to acquire the ability to proliferate and thus develop metastatic tumors. Therefore, it is believed that tumor cells with an intermediate phenotype can spread to distant sites and grow most effectively [26]. Additionally, CTCs can form aggregates with leukocytes, endothelial cells, or platelets, called CTC clusters [27]. Initially, it was hypothesized that CTC clusters arose from the co-invasion and propagation of oligoclonal populations of tumor cells [27, 28]. Nonetheless, recent studies have shown that these clusters can be formed by tumor cells aggregating in the vascular system induced by the affinity interaction of the hyaluronan receptor CD44 [29]. Compared with a single tumor cell, CTC clusters are relatively rare in circulation but exhibit remarkably greater resistance to apoptosis and more metastatic potential [30, 31]. Moreover, studies on CTC clusters in the peripheral blood of patients with CRC have shown that CTC clusters are not malignant, but rather tumor-derived endothelial cells associated with tumor vascular characteristics; notably, the isolation and counting of these CTC clusters can distinguish between healthy individuals and patients with early-stage CRC having a high degree of accuracy (≤ IIa) [32].

Precision medicine remains a realistic vision in the field of oncology. However, tumor cell heterogeneity is a huge obstacle; many factors contribute to it and affect several biological processes associated with tumor progression [33]. Therefore, detecting CTCs in the peripheral blood of patients with CRC should be considered not only as a less invasive option than actual biopsy but also as a new approach for a comprehensive understanding of the whole tumor heterogeneity. The in-depth study of CTCs may enable the unrevealing of the molecular characteristics of tumors, identification of biomarkers for targeted therapies, prediction of the effectiveness of treatment, and precise assessment of tumor prognosis, thus enabling precision medicine.

## CTC enrichment techniques

The techniques for isolating CTCs can be divided into two categories according to the inherent physical characteristics of the cells or the specific antigens expressed on the surface (Table 1). CTCs are usually identified by immunocytochemistry, immunofluorescence, or other techniques after being captured [34]. CTC separation and capture techniques have been constantly updated with increasing efficiency and purity in recent years.

**Table 1:** CTC detection technologies

	Subcategory	Key features	Capture yield	Reference
Biophysical property				
ISET	Microfiltration	Based on the principle that CTCs differ in size from other blood cells, isolation is performed using an 8-µm microporous polycarbonate membrane filter	N/A	[35]
Parsortix PR1	Microfiltration	The system allows CTC enrichment using cell size and membrane deformability	68%	[36]
Ficoll-Paque	Density gradient centrifugation	It was not used to isolate CTCs initially; it was later used to capture CTCs in peripheral blood of various cancers and easy to be used in combination with other techniques with satisfactory results	84%	[37]
Oncoquick	Microfiltration	Porous membranes are used to reduce the number of blood cells similar to the size of CTCs	87%	[38]
DepFFF	Electrophoresis	CTCs can be separated under the action of the electric field due to the different sizes and charges of blood cells	70%	[39]
PAFC	In vivo imaging	It works by absorbing laser light through nanoparticles labeled with antibodies on the target cells and enables the real-time detection of CTCs in veins by laser technology	N/A	[40]
ScreenCell	Microfiltration	A microfluidic platform for CTC separation based on cell size difference, which uses track-etched membranes with nano- to micron-sized pores in thin polycarbonate films	74–91%	[41]
CellSieve	Microfiltration	With the help of the array of precision pores, the platform can capture CTCs in a low-pressure state and protect the intracellular structure at the same time	83–91%	[42]
FMSA	Microfiltration	CTCs can be rapidly enriched directly from whole blood, and the cell damage is reduced to a minimum with the help of flexible polymer microsprings	90%	[43]
CanPatrol	Microfiltration	Cells are microfiltered and then detected using multiplex RNA in situ hybridization for biomarkers representing the CTC phenotype	89%	[44]
Immunoaffinity				
CellSearch	Immunomagnetic	It combines the EpCAM expressing on the surface of CTCs with immunomagnetic beads containing antibodies and produces shunt under the action of a magnetic field	85%	[45]
MagSweeper	Immunomagnetic	It uses a magnetic rod to capture CTCs in vivo using the immune affinity principle and then releases CTCs in vitro, and finally obtains high-purity CTCs	86%	[46]
CTC-Chip	Microfiltration	It is a platform consisting of anti-EpCAM antibody-coated microposts, which can capture CTCs under precisely controlled laminar flow conditions without pre-labeling or pre-processing	60%	[47]
HB-Chip	Microfiltration	This technique increases the contact between CTCs and antibodies on the chip surface by generating microvortices, thus improving the capture efficiency and purity of CTCs	74–84%	[48]
CTC-iChip	Microfiltration, immunomagnetic	The device removes the cells except CTCs and blood cells through a micro-column structure and then quickly separates and captures CTCs using the immunomagnetic beads	97%	[49]
IsoFlux	Microfiltration, immunomagnetic	This platform controls the flow of cell suspensions in the microfluidic system and uses immunoaffinity to capture CTCs	N/A	[50]
AdnaTest	Immunomagnetic	This platform uses a combination of three antibodies (EpCAM, EGFR, and MUC1) for CTC capture and allows gene detection by multiplex RT-PCR gene panel	N/A	[51]

## Isolation based on physical properties

CTCs usually exhibit low density and tend to be comparatively larger in diameter compared with other blood cells [52]. A large number of platforms for CTC isolation based on physical properties have satisfying capture efficiency, which can be roughly divided into the following categories.

### Based on size

Isolation by size of epithelial tumor cells (ISET) is the platform for capturing CTCs after blood filtration based on the difference in cell size; it can capture CTCs from 1 mL of peripheral blood samples for counting and immunomorphological analysis [35]. Su et al. [53] developed a new integrated microfluidic device to isolate CTCs based on the difference in size and deformability between tumor cells and normal blood cells. CTCs were successfully captured in the peripheral blood samples of all patients with advanced CRC within 3 h. Cohen et al. [36] proposed the Parsortix PR1 system, which allows CTC enrichment based on cell size and membrane deformability; also, the expression of more than 2000 cancer-related genes can be obtained in the downstream analysis of captured CTCs using the HTG EdgeSeq nuclease protection assay. Ribeiro Samy et al. [54] developed a size- and deformability-based microfluidic chip for capturing CTCs. Moreover, the droplet digital pathologic complete response (PCR) (ddPCR) was used for the CTC molecular characterization, revealing the existence of APC gene mutations in most patients with CRC. Likewise, ScreenCell [41], CellSieve [42], and FMSA (Flexible Micro Spring Array) [43] are all microfluidic platforms that capture CTCs based on the principle of cell size difference.

### Based on a density gradient

This separation method takes advantage of the principle that cells differ in density from each other. It can cause stratification of cells, with CTCs remaining in the monocyte-enriched layer, by adding the separation medium to the blood and centrifuging it. Density gradient centrifugation is a simple, reliable, and inexpensive method. Nonetheless, the disadvantages of this method include the loss of large amounts of CTCs and the fact that leukocytes cannot be readily removed. This results in considerably low purity of obtained CTCs, which is not conducive for further downstream experimentation [55]. Ficoll-Paque was not initially used to isolate CTCs, but later, it was used to capture CTCs in the peripheral blood of cancer patients with satisfactory results [37]. Rosenberg et al. [38] proposed a device to achieve higher CTC purity, called OncoQuick, which uses porous membranes to reduce the number of blood cells with similar size to CTCs and has been shown to have a high CTC recovery rate.

### Based on inertia

The differences in the size and density of CTCs from other blood cells reflect different cell dynamics. Di Carlo et al. [56] proposed an inertial microfluidic system to isolate CTCs without antibody labeling, and the capture efficiency of the device was as high as 90%; it could process 2.5 mL of blood every minute.

### Based on electrophoresis

Gascoyne et al. [39] proposed dielectrophoretic field-flow fractionation (DepFFF) to isolate CTCs; it works on the principle that CTCs can be separated in the presence of an electric field due to the different sizes and charges of cells. Thus, the platform exploits the intrinsic properties of CTCs without adding biomarkers to the cell surface and can provide unmodified live CTCs for in vitro culture and downstream analysis [57, 58]. The shortcomings of this device include the low capture rate of approximately 70% and the low efficiency of processing the peripheral blood of approximately 1 mL per hour [59].

### Based on photoacoustic effect

Bhattacharyya et al. [60] used photoacoustic flow cytometry (PAFC) to detect CTCs in the peripheral blood of patients with breast cancer. It works by the absorption of laser light through nanoparticles labeled with antibodies on target cells. They also found that this method could be used to study the response of patients with breast cancer to treatment as well as in vitro drug trials [60].

## Isolation based on immune affinity

Besides the capture methods based on physical principles, the immunoaffinity-based capture methods take advantage of the expression of specific biomarkers on the surface of CTCs. CellSearch is a CTC capture platform developed by Veridex and approved by the Food and Drug Administration (FDA). It works by using immunomagnetic beads with EpCAM antibodies modified on the beads to capture CTCs through immunoaffinity with the EpCAM antigen expressed on the surface of CTCs and then subsequently generating a shunt under the action of a magnetic field. The captured CTCs can then be counted and immunoassayed [45]. Based on this, the CTC counts and dynamic changes may provide prognostic information in patients with cancers [45]. Recently, several studies used this platform to capture CTCs and found correlations with the poor prognosis of patients with CRC, thus demonstrating the potential of CTC as a prognostic biomarker [61–67]. Furthermore, the CTC count has been approved by the FDA as a standard for evaluating the prognosis of metastatic CRC [61], showing that liquid biopsy has a great potential for clinical application in the future. However, the CellSearch is limited by the amount of blood taken. The expression of EpCAM could be reduced due to the EMT process, resulting in the false-negative rate of this single antibody capture system. Therefore, other types of antibodies, such as N-cadherin, Vimentin, and combinations of multiple antibodies [68–70], which have immunoaffinity to cells with mesenchymal properties, have also been introduced into the CTC capture platform for capturing cells expressing low quantities of EpCAM. In addition, antibodies that do not depend on epithelial and mesenchymal properties have also been developed for CTC capture. A subunit of the VAR2CSA protein called rVAR2 was recombinantly expressed in *E. coli* by Agerbaek et al. and successfully used to capture CTCs from tumor patients [71, 72].

The development of microfluidic-based CTC capture platforms is also a hot research topic in recent years. It can use not only the difference in cell size, but also immunoaffinity, to separate CTCs, and thus has certain advantages over ordinary immunomagnetic beads. CTC-Chip [47], HB-Chip [48], and CTC-iChip [49] are a few representative capture platforms of such microfluidic systems that use immunoaffinity for CTC capture based on the principle that target cells can specifically bind to microchannels or structurally modified bioactive molecules in microfluidic devices. Although CTCs can be easily captured by combining cell surface − specific antigens with bioactive molecules via immunoaffinity, the purity of CTCs may be compromised because, in addition to white blood cells, the whole blood contains platelets, neutrophils, eosinophils, lymphocytes, and other cells, resulting in nonspecific capture [73]. To solve this problem, negative selection platforms were designed to remove non-target cells such as white blood cells and red blood cells [74]. Since the enrichment process is not affected by the expression of cell surface antigen, this type of device has a high capture efficiency and thus facilitates further downstream experiments. However, the limitation is that the purity of isolated CTCs is lower than that of positive enrichment and is prone to the loss of CTCs [75]. Although studies have suggested that CTC counting and differentiation of CTC subtypes by immunostaining may be effective in evaluating the prognosis of various cancers, a comprehensive molecular and functional analysis of CTC may better characterize the metastatic potential of tumors to develop precise treatment plans. Several capture platforms allow for downstream analysis after CTCs are isolated. For example, IsoFlux uses flow control and immunomagnetic capture to achieve high purity of CTCs for downstream analysis; also, the platform can effectively reduce the damage of CTCs during the capture process [50]. AdnaTest is a CTC capture platform that has been modified with three antibodies (EpCAM, EGFR, and MUC1), allowing for the identification of tumor-associated transcripts by RT-PCR [51]. As of now, many studies have used this category of platforms for CTC capture and downstream analysis to investigate the association between gene expression and cancer prognosis [76–78]. Nowadays, with the rapid development of nanotechnology, nanomaterials have been successfully introduced into the study of CTC isolation. The basic principle is based on the strong interaction between CTCs and nano-substrates, such as nanocolumns, nanofibers, nanoparticles, nanotubes, and nanorough surfaces, all of which exhibit satisfactory capture sensitivity and efficiency [79].

## Clinical applications of CTCs

Traditional diagnostic and staging detection strategies for CRC have many limitations, resulting in an inadequate understanding of disease progression. Therefore, the exploration of representative biomarkers is urgently needed for early diagnosis, recurrence prediction, and prognostic evaluation of patients with CRC. A wealth of information on primary tumor prediction, disease progression, and prognostic follow-up can be obtained by detecting CTCs in peripheral blood (Table 2).

**Table 2:** Clinical application of CTCs in colorectal cancer

Study	Patients	Method	CTC marker		Main finding	Ref
Early diagnosis						
Tsai et al	667 adults	CellMax	CK20, CD45, and DAPI		A significant association was found between CTC counts and worsening disease stage; besides, healthy participants could be distinguished from patients with adenomas	[80]
Baek et al	74 patients and 3 healthy volunteers	Fast	EpCAM, CD45, and DAPI		Vascular invasion was often found in patients with a CTC level ≥ 5/7.5 mL, and CTC counts of patients with stage IV were positive; besides, patients with a CTC level ≥ 5/7.5 mL showed a trend of poor overall survival and no progress	[81]
Prognosis evaluating						
Cohen et al	430 patients	CellSearch	EpCAM, CD45, and DAPI		Patients with a baseline CTCs ≥ 3/7.5 mL had shorter median PFS and OS than the patients with CTCs < 3/7.5 mL	[61]
Wang et al	121 patients	Cyttel, negative enrichment	CD45 and DAPI		The CTC positive rate was correlated with the depth of tumor invasion, lymphatic metastasis, distant metastasis, TNM stage, and serum CEA level. The multivariate Cox regression analyses revealed that the existence of CTCs during chemotherapy was an independent factor for the poor PFS and OS in patients with advanced CRC	[82]
Yang et al	138 patients	ISET	Wright’s staining		The positive rate of preoperative CTC was positively correlated with tumor stage and serum CEA level, while the positive rate of postoperative CTCs was positively correlated with tumor stage, and independently correlated with tumor recurrence-free survival rate (RFS). Postoperative, but not preoperative, CTC positive rate was an independent indicator of poor prognosis for patients with CRC treated with curative resection	[83]
Bahnassy et al	63 patients with NMCRC, 40 with benign bowel diseases, and 40 normal controls	Flowcytometry CellSearch	CK19, MUC1, CD44, CD133, and ALDH1		The positive rate of CTCs and mRNA markers was significantly correlated with short 5-year PFS and OS of NMCRC. A CTC level ≥ 1/7.5 mL was significantly correlated with the decrease in OS in patients with NMCRC	[67]
Dizdar et al	80 patients	GILUPI CellCollect (CC) CellSearch (CS)	panCK, EpCAM, CD45, and DAPI		In M0 patients, the frequency of CTCs detected by CC was higher than that by CS. In addition, no significant correlation was found between CTCs detected by CC and clinicopathological parameters or overall survival	[84]
Bidard et al	153 patients	CellSearch	panCK, EpCAM, CD45, and DAPI		The baseline CTC ≥ 3 was an independent prognostic factor for OS in patients with colorectal cancer in multivariate analysis	[85]
Zhang et al	98 patients	Size-based platform	panCK, anti-vimentin, anti-CD45, and Hoechst		CTCs were positively correlated with tumor de-differentiation, lymphatic invasion, TNM stage, and serum CEA level. PFS and OS were significantly worse in CTC positive patients with advanced CRC	[86]
Nicolazzo et al	84 patients	CellSearch	CD45, vimentin, DAPI, EpCAM, and CK20		CTCs isolated from patients with right CRC showed a distinct epithelial-like phenotype (EpCAM + , ck20 + , vimentin − /N-cadherin −), whereas CTCs from patients with left CRC showed a mesenchymal-like phenotype (EpCAM + / − , ck20 − , vimentin + /N-cadherin +) and had a worse prognosis	[87]
Virginia et al	44 patients	CellSearch	EpCAM, CD45, and DAPI		In patients with liver metastases from colorectal cancer, preoperative CTC > 2 was associated with disease progression and poor survival even though the tumor was completely resected	[88]
Chen et al	90 patients and 151 controls	Nested PCR	CK19 mRNA		The expression of ECT2 was higher in peripheral blood of patients with advanced colorectal cancer, which indicated that ECT2 might be used to predict tumor stage and clinical prognosis	[89]
Kim et al	70 patients	CD45 depletion	CD45		A total of 70 patients with colorectal cancer were classified according to T staging. The positive expression rates of MAGE A1-6 and hTERT in T3 and T4 patients were significantly higher than those in T1 and T2 patients	[90]
Ning et al	78 patients and 20 controls	CD45 depletion	CD45		The positive expression of CTC Akt-2 indicated shorter PFS and OS; therefore, it could be used to evaluate the prognosis of mCRC	[91]
Hinz et al	299 patients	Nested PCR	EpCAM and CK		The expression of CTC CK20 in peripheral blood of patients with colorectal cancer was a highly specific and independent prognostic marker	[92]
Messaritakis	436 patients	Gradient density centrifugation	N/A		CEACAM5 mRNA-positive CTCs in peripheral blood of patients with metastatic colorectal cancer predicted poor prognosis, especially in patients with KRAS and BRAF mutations	[93]
Cai et al	90 patients and 151 controls	CanPatrol	panCK, EpCAM, Vimentin, Twist, CD45, and DAPI		COX-2 might be related to the formation of colorectal cancer, and the high expression of COX was a high-risk factor for the malignant transformation of polyps	[94]
Wang et al	66 patients	CanPatrol	panCK, EpCAM, Vimentin, Twist, CD45, and DAPI		The expression level of LGR5 increased with the development of CRCr metastasis, but no significant association was observed between LGR5 expression and survival	[95]
Treatment monitoring						
Satelli et al	101 patients	EasySep		EpCAM, SLUG, E-Cadherin, β-catenin, c-myc, FOXC2, TWIST-1, SNAIL	Patients with CTC > 5/mL were more likely to resist treatment and exhibit progressive disease, while patients with CTC < 5/mL were more likely to be treatment sensitive and exhibit stable disease. In addition, CTCsCSV + might represent a distinct subpopulation of CTCs that are drug resistant and therefore insensitive to chemotherapy	[96]
Shi et al	55 patients	Gradient density centrifugation		N/A	The expression of CEA, EP-CAM, CK18, and CK19 and the number of CTC decreased with time after cryotherapy in patients with liver metastasis of colorectal cancer	[97]
Delgado-Urena et al	77 patients	Carcinoma Cell Enrichment and Detection kit		panCK, VEGFR	The decrease in the positive rate of CTCs and CTCVEGFR + in the group with good response to chemotherapy indicated that dynamic detection of CTCs could help monitor the treatment	[98]
Yue et al	35 patients	Pep@MNPs-biotinEpCAM		CK19, DAPI, CD45, and PD-L1	The number of CTCsPD-L1 + at baseline could be used as a biomarker to predict the treatment response to PD-1/PD-L1 treatment	[99]
Troncarelli Flores et al	30 patients	ISET		CD45 and DAB	Detection of TYMS and RAD23B expression in CTCs could strongly predict the response to treatment in patients with colorectal cancer. In addition, detection of TYMS mRNA in CTCs might be a valuable tool for identifying nonresponsive patients before NCRT	[100]
Huang et al	2388 patients	Meta-analysis		N/A	Patients with CRC who converted from CTC-high to CTC-low or who continued to have CTC-high during treatment had a worse prognosis than those who converted from CTC-high to CTC-low	[101]

### Early diagnosis

Tsai et al. [80] used the self-developed platform called CellMax to isolate and subsequently count CTCs from the peripheral blood of 667 Taiwanese adults who underwent enteroscopy. A predefined algorithm was then used for early diagnosis. The result showed that the platform had high specificity (86%) and sensitivity (95%) to distinguish between benign and malignant tumors. This also indicated that CTC detection had a good application prospect in the early diagnosis of CRC. Baek et al. [81] set the cutoff value of CTCs to 5/7.5 mL and used this as a criterion to differentiate between patients with CRC and healthy people with good sensitivity (75%) and specificity (100%). The results of these two studies showed the promising application of CTC detection in the early diagnosis of CRC. However, uniform criteria for differentiating benign from malignant tumors are still lacking.

### Prognosis evaluation

Several relevant studies have demonstrated a strong correlation of CTC counts with cancer progression and prognosis [102–105]. Additionally, FDA has approved the use of CTC counting as a prognostic tool for metastatic prostate, colon, and breast cancers [61]. In a prospective multicenter study involving 430 patients with mCRC, the baseline CTCs were counted using immunomagnetic beads. Then, the patients were divided into positive and negative groups according to the cutoff value of 3/7.5 mL. The result showed that the median progression-free survival (PFS: 4.5 months vs 7.9 months; *P* = 0.0002) and the overall survival (OS: 9.4 months vs 18.5 months; *P* = 0.0001) was shorter in the positive group than in the negative group [61]. A recent study of 121 patients with CRC found that 71 patients were positive for baseline CTCs, and the positivity rate highly correlated with the depth of tumor invasion, lymphatic metastasis, distant metastasis, TNM stage, and serum CEA levels [51]. Moreover, the Kaplan–Meier survival curve showed that PFS (14 months vs 23 months, *P* = 0.001) and OS (18 months vs 25 months, *P* = 0.003) of baseline CTC-positive patients were significantly shorter than those of negative patients [82]. Yang et al. [83] proposed that the positive rate of preoperative CTCs in CRC positively correlated with the tumor stage and serum CEA level while the positive rate of postoperative CTCs positively correlated with the tumor stage and independently correlated with the tumor recurrence-free survival (RFS) rate. Additionally, the study demonstrated that postoperative CTC positivity was an independent indicator of poor prognosis in patients with CRC [76]. Moreover, the risk of recurrence was higher in postoperative CTC-positive patients than in preoperative CTC-positive patients [83]. Many studies have confirmed that the CTC count is an independent factor for PFS and OS in CRC [67, 84–87]. However, Le et al. [106] performed CTC isolation from the pulmonary venous blood of 24 patients with CRC treated with pulmonary metastasectomy. The results revealed no significant correlation of PFS and OS with CTC counts. Although CTC counts have attracted widespread interest as a useful marker for cancer prognosis, they are still not widely available for clinical application. Moreover, uniform cutoff values for assessing the prognosis and progression of cancer are still lacking. Therefore, studies have been conducted to investigate the genes expressed by CTCs and correlate their expression with the clinicopathological features of cancers [78, 107–109]. Chen et al. [82] reported that the epithelial cell transforming sequence 2 (ECT2) oncogene expressed by CTCs in peripheral blood had higher sensitivity than the serum CEA level; it was highly expressed in patients with advanced-stage CRC. Furthermore, the ECT2 gene expressed on the surface of CTCs can be used as a marker for the prognosis of patients with CRC [89]. Another study including 70 patients with CRC showed that the expression level of MAGEA1-6 or hTERT genes in CTCs was significantly higher in patients with T3 and T4 stages than in patients with T1 and T2 stages [90]. Ning et al. [110] showed that the expression of the Akt-2 gene in CTCs of patients with CRC predicted shorter PFS and OS; also, the median survival time of patients with CTC^Akt−2+^ was significantly shorter than that of patients with CTC^Akt−2−^. Nowadays, the expression of CK20 [92], CEACAM5 [93], COX-2 [94], and LGR-5 [95] has also been used to monitor the progression and prognosis in patients with CRC.

### Treatment monitoring

CRC is overly aggressive and prone to metastasis. Hence, the selection of the most appropriate treatment and the best time for patients is highly challenging. Therefore, reliable biomarkers for accurate monitoring of the treatment effect are urgently required. Additionally, CTC subclones can reflect the heterogeneity of cancers in real time and show their different abilities to evade treatment; thus, CTCs can be monitored dynamically to understand the response to treatment in time [98]. Shi et al. [97] proposed that the number of CTCs in the peripheral blood of patients with liver metastases from CRC significantly reduced 7 and 30 days after cryotherapy. Moreover, the expression of serum CEA, EpCAM, CK18, and CK19 gradually decreased with time. Delgado-Urena et al. detected the CTCs of 77 patients with mCRC treated with FOLFOX and bevacizumab before treatment and 12 and 24 weeks after treatment. The patients were divided into good response group (complete response group, partial response group, or stable group) and poor response group. The final results revealed that the positive rates of CTCs and CTC^VEGFR+^ decreased in the group with good treatment response [98], suggesting that the dynamic detection of CTCs could monitor the treatment in a timely manner, allowing physicians to understand the treatment response and change the treatment regimen early. In another study of 35 patients with gastrointestinal cancer, Yue et al. [99] reported that CTCs with high expression of PD-L1 could be used as a biomarker to predict the PD-1/PD-L1 treatment response. In yet another study of preoperative neoadjuvant therapy for CRC, Troncarelli Flores et al. reported that the baseline CTCs ^TYMS+/ RAD23B+^ were associated with poor reactions of neoadjuvant chemoradiation (NCRT) therapy. CTCs ^TYMS−/RAD23B−^ were detected in all PCR patients after neoadjuvant therapy, 83.5% of partial response (PR) patients, but not in no response (NR) patients; in comparison, CTCs^Tyms+/RAD23B+^ were not detected in PCR or PR patients but could be detected in 83.5% of NR patients [100].

### Culture of CTCs in vitro

The establishment of permanent CTC cell lines in vitro has been one of the challenging studies in recent times. Indeed, CTC cell lines can be used to identify proteins and pathways associated with cancer cell stemness and metastasis, as well as to test the sensitivity of cancers to anti-cancer drugs [111]. Cayrefourcq et al. [112] successfully cultivated a CTC cell line isolated from a patient with colon cancer named CTC-MCC-41, which had been cultured for more than 1 year by the time the study was published. The cell line has been characterized at the genome, transcriptome, proteome, and secretome levels. More importantly, functional studies revealed that the CTC cell line could quickly form tumors after transplantation in immunodeficient mice. Pantel et al. performed a follow-up study on the CTC-MCC-41 cell line and found that CTC-MCC-41 had the following characteristics besides the ability to expand in vitro for more than 2 years: (A) stem cell-like epithelial characteristics, (B) epithelial/mesenchymal phenotype, (C) potential for rapid initiation of angiogenesis in vitro, and (D) bone-like characteristics. Moreover, the cell line had characteristics similar to those of primary tumors and metastatic lymph nodes in patients with CRC [113]. De et al. [114] proposed a scheme for the 3D culture of CTCs in vitro, which used a polycaprolactone scaffold to deposit an extracellular matrix on it. The study also confirmed the existence of a CTC mixed phenotype, thus helping to better understand the relationship between CTC phenotype and CRC prognosis. Grillet et al. [115] conducted downstream experiments after culturing CTCs of patients with CRC in vitro. The results showed that the patients who successfully established CTC-MCC-411 died soon after receiving chemotherapy with the FOLFIRI regimen. The drug sensitivity test also revealed that the cell line established in vitro was resistant to the FOLFIRI regimen. Notably, the results revealed significant overexpression of genes associated with xenograft resistance in their cultured CTC lines. Cytotoxicity assays further confirmed the potential use of the model in predicting drug response in patients with CRC.

### CTC single-cell analysis

TC single-cell analysis. Tumor cells have a high degree of heterogeneity at the single-cell level, which may be one of the factors responsible for increasing the incidence of cancer recurrence and metastasis [33, 116]. Single-cell technology can help unravel the heterogeneity of tumors. Therefore, an in-depth analysis of a single CTC allows the real-time monitoring of somatic mutation profiles and genomic expression of drug-resistant clones in patients with cancer, contributing to a deeper understanding of the treatment of human cancers such as CRC [33, 117]. Comparing a single CTC with primary and metastatic tumors may help identify the clones that cause metastasis and reveal the molecular biomarkers of metastasis [118]. Single-cell RNA sequencing (scRNA-seq) can be used as a powerful tool to discover new tumor progression markers in CTCs. Recent studies have reported the genomic and transcriptomic analyses of single CTC using next-generation sequencers [118, 119]. Abouleila et al. [6] reported a method of combining single-cell mass spectrometry with microfluidic cell enrichment and obtained a nontargeted molecular map of a single CTC from the peripheral blood of patients with gastrointestinal cancer. This revealed the differences in metabonomic characteristics among CTCs from different tumor populations. Pei et al. [120] reported an integrated microfluidic device for the phenotypic analysis of a single CTC. This device can allow the automatic isolation of CTCs from whole blood after sequential single-cell phenotyping with high-purity (92% ± 3%) cell sorting and high-throughput processing capacity (5 mL/3 h). This study reported the collection of a single CTC from xenografted mice and patients with different stages of CRC and obtained the correlations of CTC characteristics with clinical tumor stage and treatment response. Single-cell transcriptomics can detect the expression levels of a single cell in a given population, and hence the interest in this area of research has increased [121]. Bian et al. [122] established a platform called the single-cell triple omics sequencing (SCTrio-seq) technique. This platform is capable of detecting gene mutations, transcription, and methylation simultaneously at the single-cell level. Ten patients with CRC underwent SCTrio-seq. The results provided insight into tumor evolution and linked DNA methylation to genetic genealogy. Additionally, the results confirmed that DNA methylation levels were consistent within the genealogy but might differ significantly between clones [122]. Using single-cell analysis techniques, it is now possible to deconstruct cancers into their constituent cell types and thus enable the understanding of the biological characteristics of cancer. The analysis of CTC gene expression patterns of CRC at the single-cell level can help find key information for evaluating prognostic biomarkers and developing precise and personalized cancer therapies.

## ctDNA analysis in CRC

Plasma ctDNA refers to DNA fragments of tumor origin and is a subset of plasma cell–free DNA (cfDNA). The cfDNA also includes DNA from other sources in the circulation, mainly germline DNA due to hematopoietic cell death [123, 124]. Hence, figuring out the origin of cfDNA may be a challenge. Besides, the shedding of ctDNA is extremely varied; it may be as little as 0.01%, or it might account for the majority of total plasma cfDNA [125]. Studies demonstrated that the amount of ctDNA was primarily associated with tumor burden and tumor type [126]. From a heterogeneous viewpoint, ctDNA offers a more comprehensive overview of the range of mutations present in a patient’s tumor [127]. Originally, the real-time quantitative PCR (qPCR) detection system was used to identify ctDNA-specific mutations [128]. However, limited by its sensitivity and specificity, PCR can only be performed in patients with advanced cancer having high ctDNA levels [129]. In contrast, digital PCR (dPCR) has a higher sensitivity in detecting and quantifying ctDNA and is currently widely used in bodily fluid biomarker studies [128, 130]. Still, one limitation of PCR-based techniques is that they cannot be used to investigate a wide range and diverse types of genomic alterations [131]. With the development of RNA sequencing technology, next-generation sequencing (NGS) has overcome these shortcomings and can detect copy number alterations, mutations, and other chromosomal rearrangements, including inversions, translocations, and reversals [132]. The recent growth in the studies demonstrates the value of ctDNA in the early diagnosis [133, 134], prognosis [135, 136], and response to treatment [137, 138] in CRC.

Compared with ctDNA, which is extensively fragmented and compounded by significant background, CTCs are intact cells providing more genetic information about the origin of tumors and potentially allowing for more specific diagnosis and more precise treatment plans for patients with CRC [139].

## Summary and future prospects

Thanks to the continuous advancements in diagnostic and treatment technologies, the prognosis of patients with CRC has improved remarkably in recent decades. Nonetheless, the early diagnosis, treatment monitoring, and prognostic evaluation of CRC still have some limitations; therefore, a large number of patients with CRC still die every year. At present, a variety of sensitive, high-throughput, and efficient CTC isolation platforms have been successfully established, which can be used to count CTCs in the peripheral blood of patients with CRC dynamically. Additionally, genes and proteins expressed by CTCs can be detected to evaluate the prognosis and recurrence of CRC. However, a uniform CTC cutoff value for clinical assessment of CRC progression and prognosis still lacks due to the differences in sampling, storage time, and enrichment methods. The downstream analysis of CTCs in patients with CRC and the single-cell technology have also improved the understanding of tumor formation, development, metastasis, and heterogeneity, which is conducive to the development of a personalized drug detection platform. Despite several CTC detection methods, the application of CTCs in clinical practice is not yet widespread due to the lack of a standardized detection platform with uniform high sensitivity and specificity. Most of the studies are single-center studies with a small number of cases. This has eventually led to many studies with different results due to interindividual differences, such as pathogenic factors, tumor stage, ethnicity, and geographical factors, to name a few. Against this backdrop, an important research direction for the future is to include a large number of cases and conduct multicenter prospective studies. Besides the number of CTCs and expressed genes or proteins as the criteria for cancer diagnosis and prognosis, CTCs in combination with other indicators such as CA-199 or ctDNA are worth investigating because they can increase the accuracy of diagnostic and prognostic assessments [69, 140]. Tumor immunotherapy has recently attracted much attention and is one of the hot spots in the field of tumor treatment. The expression of immune checkpoints on the surface of CTCs is also associated with the effect of immunotherapy. Therefore, the blocking of these checkpoints can be used as an immunotherapeutic approach. Lian et al. successfully targeted PD-L1 and CD47 on the surface of CTCs, which eventually caused a significant inhibition of tumor growth and metastasis [141]. Although CTCs are not prevalently used and promoted in clinical practice, their role in tumor prognostic assessment cannot be ignored. Therefore, one of the future research directions should be to develop a CTC capture platform with higher efficiency, specificity, and sensitivity. Additionally, more comprehensive and in-depth studies of CTCs from a new perspective must be conducted to develop a more personalized and precise treatment plan for each cancer patient.
